# Early and Increased Influenza Activity Among Children — Tennessee, 2022–23 Influenza Season

**DOI:** 10.15585/mmwr.mm7203a1

**Published:** 2023-01-20

**Authors:** Christine M. Thomas, Elizabeth B. White, Noah Kojima, Mary-Margaret A. Fill, Samir Hanna, Timothy F. Jones, Caitlin N. Newhouse, Kelly Orejuela, Emma Roth, Sarah Winders, Daniel R. Chandler, Carlos G. Grijalva, William Schaffner, Jonathan E. Schmitz, Juliana DaSilva, Marie K. Kirby, Alexandra M. Mellis, Melissa A. Rolfes, Kelsey M. Sumner, Brendan Flannery, H. Keipp Talbot, John R. Dunn

**Affiliations:** ^1^Epidemic Intelligence Service, CDC; ^2^Tennessee Department of Health; ^3^Influenza Division, National Center for Immunization and Respiratory Diseases, CDC; ^4^Vanderbilt University Medical Center, Nashville, Tennessee.

Influenza seasons typically begin in October and peak between December and February (*1*); however, the 2022–23 influenza season in Tennessee began in late September and was characterized by high pediatric hospitalization rates during November. This report describes a field investigation conducted in Tennessee during November 2022, following reports of increasing influenza hospitalizations. Data from surveillance networks, patient surveys, and whole genome sequencing of influenza virus specimens were analyzed to assess influenza activity and secondary illness risk. Influenza activity increased earlier than usual among all age groups, and rates of influenza-associated hospitalization among children were high in November, reaching 12.6 per 100,000 in children aged <5 years, comparable to peak levels typically seen in high-severity seasons. Circulating influenza viruses were genetically similar to vaccine components. Among persons who received testing for influenza at outpatient clinics, children were twice as likely to receive a positive influenza test result as were adults. Among household contacts exposed to someone with influenza, children were more than twice as likely to become ill compared with adults. As the influenza season continues, it is important for all persons, especially those at higher risk for severe disease, to protect themselves from influenza. To prevent influenza and severe influenza complications, all persons aged ≥6 months should get vaccinated, avoid contact with ill persons, and take influenza antivirals if recommended and prescribed.*

The field investigation was conducted in November 2022 to understand early influenza activity in 14 of 95 Tennessee counties clustered in middle Tennessee and to identify groups most affected.^†^ Weekly, age group–stratified data on emergency department visits for influenza-like illness (ILI-ED) and influenza-associated hospitalizations were obtained from the Electronic Surveillance System for the Early Notification of Community-Based Epidemics (ESSENCE) and FluSurv-NET surveillance systems,^§^ respectively, and were compared with data from previous influenza seasons.^¶^ For influenza-associated hospitalizations, a probability distribution constructed from the three highest weekly rates from each previous season (*2*) was used to define age group–specific intensity thresholds for the 50th (medium), 90th (high), and 98th (very high) percentiles. Hospitalization data from previous seasons were adjusted for underdetection using data on age group–specific testing practices; 2022–23 season data were not adjusted. All surveillance data were restricted to October 2, 2022–January 7, 2023, reported as of January 12, 2023.

Vanderbilt University Medical Center (VUMC) and the Mid-Cumberland Regional Health Department clinics provided the Tennessee Department of Health (TDH) with information about persons who received testing for influenza. These data were merged with influenza vaccination records for the current season obtained from the Tennessee Immunization Information System. Persons who received a positive or negative influenza test result during November 4–18, 2022, were invited to complete a survey asking about their illness, which was facilitated by REDCap electronic data capture tools hosted at TDH** (*3*,*4*). Those who received a positive influenza test result were invited to complete a second, follow-up survey 1 week after their test to inquire about illnesses in household contacts.^††^ VUMC clinics provided influenza-positive specimens collected during November 4–18 to CDC for whole genome sequencing to characterize circulating influenza viruses.

Factors associated with positive influenza test results in patients who received testing at participating clinics were identified using logistic regression. Characteristics of household contacts were compared using logistic regression accounting for household clustering. Secondary attack rates for symptomatic illness among household contacts with adjustment for age, vaccination status, and household size were assessed with a chain binomial model, assuming a 2-day infectiousness period, beginning at symptom onset, and a 2-day incubation period (*5*). Descriptive and regression analysis of outpatient data was conducted using SAS software (version 9.4; SAS Institute), and the chain binomial modeling was conducted in C. This activity was reviewed by CDC, TDH, and VUMC and was conducted consistent with applicable federal law and CDC policy.^§§^

During October 2, 2022–January 7, 2023, ILI-ED visits and influenza-associated hospitalization rates began increasing and reached high levels earlier than in recent influenza seasons. These trends were most evident among persons aged <18 years, with ILI-ED visits in this group accounting for 31% of all visits during the week ending November 26, 2022 (previous seasons’ peak levels range = 14%–34%). Weekly pediatric influenza-associated hospitalizations reached 12.6 per 100,000 children aged <5 years and 6.9 per 100,000 persons aged <18 years, exceeding the 90th percentile (high intensity) and approaching the 98th percentile (very high intensity) of peak weekly rates for children reported during previous influenza seasons (Figure). In comparison, among persons of all ages, ILI-ED reached 15% of all visits (previous seasons’ peak range = 5%–13%), and influenza-associated hospitalizations surpassed medium intensity.

**FIGURE:**
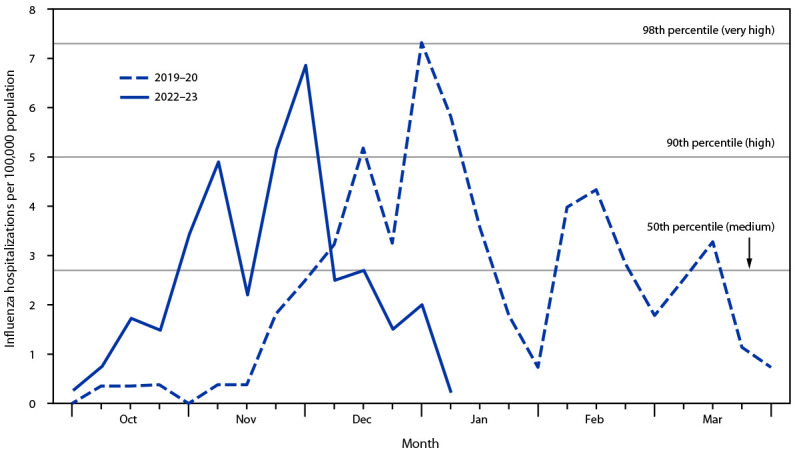
Influenza-associated hospitalizations and influenza intensity thresholds among persons aged <18 years — Tennessee, 2019–20 and 2022–23 influenza seasons* * Medium, high, and very high thresholds are based on the probability distribution of peak weekly influenza-associated hospitalization rates for persons aged <18 years in Tennessee in the nine most recent seasons. The 2019–20 season is also shown for comparison because it had the highest peak rate for this age group in Tennessee in recent seasons.

Among 4,626 persons from participating outpatient clinics who received influenza testing during November 4–18, 2022, a total of 2,164 (47%) were children, who were more likely to receive a positive test result (33%; 714 of 2,164) than were adults (20%; 483 of 2,462) (p<0.001). Seasonal influenza vaccination coverage was low in both children (23%; 499 of 2,164) and adults (34%; 830 of 2,462) who were tested for influenza. Among 332 specimens with completed sequencing data, 179 (54%) were classified as A(H3N2)3C.2a1b2a.2, and 153 (46%) as A(H1N1)pdm096B.1A5a.2; all were genetically similar to vaccine components.^¶¶^ Among 489 persons who responded to the survey (11% of 4,626 patients contacted for the survey), 269 (55%) were reported as children. Among 238 persons surveyed who had a positive influenza test result within 48 hours of symptom onset, 109 (46%) received influenza antiviral medication. Children were less likely to be treated (41%; 63 of 155) than were adults (55%; 46 of 83) (p = 0.03).

One hundred eighty-five persons with influenza completed the follow-up household survey.*** Among their 480 household contacts, 151 (31%) reported having any illness before or after the respondent’s illness, among whom 83 (55%) reported symptoms of fever and either cough or sore throat consistent with influenza-like illness. From a univariate logistic regression model, household contacts reporting any illness were more likely to be children (odds ratio [OR] = 1.85; 95% CI = 1.45–2.35) or share a bedroom with an ill person (OR = 2.11; 95% CI = 2.59–2.80) during the week before or after the respondent’s positive influenza test date compared with contacts who were not ill. In the transmission model, children were more likely to become ill than were adults (adjusted OR = 2.50; 95% CI = 1.55–4.03) (Table). After adjustment for household size and reported vaccination status, the secondary attack rate was 11.9% (range = 6.1%–22.0%) among child contacts and 5.1% (range = 2.7%–9.4%) among adult contacts.

**TABLE:** Characteristics of 480 household contacts of 185 persons who received a positive influenza test result and percentage of contacts who reported illness compatible with influenza — Tennessee, November 2022

Characteristic	Ill contacts/Total contacts (%)	Adjusted OR (95% CI)*
**Age group, yrs**		
<18	76/170 (45)	2.5 (1.6–4.0)^†^
≥18	75/310 (24)	Ref
**Vaccination status**		
Unvaccinated	103/306 (34)	Ref
Vaccinated	48/174 (28)	1.0 (0.6–1.6)
**Total household size**		
<5 persons	77/277 (28)	Ref
≥5 persons	74/203 (36)	1.2 (0.5–2.6)

**Abbreviations:** OR = odds ratio; Ref = referent group.

* Adjusted ORs of illness were estimated using a chain binomial model accounting for age, vaccination status, and household size. In this model, age and vaccination status were covariates affecting susceptibility to illness from within the household and in the community, and household size was a covariate affecting susceptibility to illness from within the household only. Covariates were chosen based on completeness; information on sharing a bedroom was missing for all index patients, and information on sex was missing for 17 persons.

^†^ Statistically significant.

## Discussion

Influenza activity in Tennessee during the 2022–23 season began earlier than in previous seasons, and the percentage of outpatient visits for influenza and the rate of influenza-associated hospitalization among children have been high. This investigation found that children seeking outpatient care were more likely to receive a positive influenza test result than were adults, and that pediatric influenza-associated hospitalization rates in late November reached levels similar to peak activity during recent high-severity influenza seasons. Together with the finding that, among household contacts, secondary illnesses occurred more frequently in children than in adults, these results suggest that children are experiencing an increased impact of influenza during the 2022–23 season.

 Influenza activity appears to have been declining in Tennessee and nationally since early December 2022; however, influenza continues to circulate and several weeks of the season remain (*6*). Although anyone can be infected with influenza viruses, young children, older adults, and persons with certain underlying medical conditions are at increased risk for influenza-associated morbidity and mortality (*7*). Seasonal influenza vaccination remains the best way to protect against influenza and influenza-associated complications, including among children. All persons aged ≥6 months should receive a seasonal influenza vaccine to protect themselves for the remainder of the influenza season. In addition, everyday preventive actions, such as reducing interactions with persons who are ill; avoiding others when ill; avoiding touching one’s mouth, eyes, and nose; frequent handwashing; and wearing facemasks when respiratory virus circulation is high, can help reduce the risk for becoming infected with influenza and other respiratory viruses.

The rates of influenza-associated hospitalization in Tennessee, especially among children, were higher during October–November 2022 than during the same months in recent influenza seasons. The hospitalization rates among children in Tennessee are similar to those seen nationally among children (*8*). Influenza antiviral medication should be prioritized for hospitalized persons and all patients at increased risk for influenza complications (including children aged <2 years and persons with certain underlying medical conditions); these medications can reduce the risk for influenza-associated complications (*9*).

The findings in this report are subject to at least five limitations. First, these findings are limited in generalizability because the population represents a sample from middle Tennessee recruited over a period of 2 weeks in November, and only 11% of invited participants completed the survey. Second, this early season assessment might not be indicative or predictive of the remainder of this influenza season. Third, because Tennessee does not require reporting to the state immunization registry, vaccination status might be underestimated. Fourth, survey participants were recruited from among clinic patients and likely represent a subset of the population with more access to health care services. Finally, the transmission model assumes that ill household contacts had influenza, which might overestimate infection because other respiratory viruses were also circulating.

In Tennessee, the 2022–23 influenza season has been marked by early and intense activity, particularly affecting children, with higher rates of pediatric influenza-associated hospitalizations than have been reported in recent influenza seasons. As the influenza season continues, it is important for all persons, especially those at higher risk for severe disease, to protect themselves from influenza. To prevent influenza and severe influenza complications, all persons aged ≥6 months should get vaccinated, avoid contact with ill persons, and take influenza antivirals if recommended and prescribed.

**Summary**

**What is already known about this topic?**

During fall 2022, many states reported increased respiratory virus activity earlier than is typically observed. Information was limited about the impact of early influenza activity.

**What is added by this report?**

After several low-severity influenza seasons, the 2022–23 season in Tennessee has been characterized by earlier activity, higher rates of pediatric hospitalization, and a higher rate of symptomatic illness among children than among adults or during past seasons.

**What are the implications for public health practice?**

To prevent influenza and severe influenza complications, all persons aged ≥6 months should get vaccinated, avoid contact with ill persons, and take influenza antivirals if recommended and prescribed.

## Footnotes

*
https://www.cdc.gov/flu/prevent/prevention.htm

† FluSurv-NET data identifies influenza-associated hospitalizations in eight middle Tennessee counties: Cheatham, Davidson, Dickson, Robertson, Rutherford, Sumner, Williamson, and Wilson. The Electronic Surveillance System for the Early Notification of Community-Based Epidemics (ESSENCE) captures emergency department visits statewide; analysis was restricted to the same eight counties. Survey data were collected from two clinic networks in middle Tennessee, including the same eight counties as well as Houston, Humphreys, Maury, Montgomery, Stewart, and Trousdale counties.

§ ESSENCE is a syndromic surveillance system that captures weekly percentages of ED visits with an influenza-specific diagnosis code or chief complaint suggesting influenza-like illness. FluSurv-NET is a population-based surveillance system that identifies weekly rates of influenza-associated hospitalizations within the catchment area.

¶ For ESSENCE data, comparison seasons included 2017–18 through 2019–20 and 2021–22. For FluSurv-NET data, comparison seasons included 2012–13 through 2019–20 and 2021–22. The 2020–21 influenza season was excluded from both comparisons because of minimal influenza activity.

** The survey was provided in the region’s most frequently spoken languages (Arabic, English, or Spanish) and facilitated by REDCap electronic data capture tools hosted at TDH with a Twilio application programming interface. Survey participants could access the survey online through a link provided by text message to their mobile telephone. In addition, TDH- and VUMC-affiliated employees called invited participants and offered the survey by telephone.

†† The follow-up survey asked about sex, age, and influenza vaccination status of household members, whether they slept in the same room as an ill person, and whether they were ill the week before or after the participant received influenza testing. If an ill household member was reported, additional questions were asked about onset date and symptoms, including fever, chills, cough, sore throat, runny nose, diarrhea, vomiting, and body aches. Responses from participants whose household members had already participated were excluded.

§§ 45 C.F.R. part 46, 21 C.F.R. part 56; 42 U.S.C. Sect. 241(d); 5 U.S.C. Sect. 552a; 44 U.S.C. Sect. 3501 et seq.

¶¶
https://www.cdc.gov/flu/weekly/

*** The median household size was three persons (IQR = three to four persons). Most (77%; 142) households included children, with a median of two children per household (IQR = one to two children).
